# Chemical and pathogen-induced inflammation disrupt the murine intestinal microbiome

**DOI:** 10.1186/s40168-017-0264-8

**Published:** 2017-04-27

**Authors:** Mikayla A. Borton, Anice Sabag-Daigle, Jikang Wu, Lindsey M. Solden, Bridget S. O’Banion, Rebecca A. Daly, Richard A. Wolfe, Juan F. Gonzalez, Vicki H. Wysocki, Brian M. M. Ahmer, Kelly C. Wrighton

**Affiliations:** 1 Department of Microbiology, The Ohio State University, 484 W. 12th Avenue, 440 Biological Sciences Building, Columbus, OH 43210 USA; 2 Department of Microbial Infection and Immunity, The Ohio State University, Columbus, OH 43210 USA; 3 Center for Microbial Interface Biology, The Ohio State University, Columbus, OH 43210 USA; 4 Department of Chemistry and Biochemistry, The Ohio State University, Columbus, OH 43210 USA

**Keywords:** *Salmonella*, Short-chain fatty acids, Inflammation, Beta diversity, LEfSe, CBA/J, Lipocalin-2

## Abstract

**Background:**

*Salmonella* is one of the most significant food-borne pathogens to affect humans and agriculture. While it is well documented that *Salmonella* infection triggers host inflammation, the impacts on the gut environment are largely unknown. A CBA/J mouse model was used to evaluate intestinal responses to *Salmonella*-induced inflammation. In parallel, we evaluated chemically induced inflammation by dextran sodium sulfate (DSS) and a non-inflammation control. We profiled gut microbial diversity by sequencing 16S ribosomal ribonucleic acid (rRNA) genes from fecal and cecal samples. These data were correlated to the inflammation marker lipocalin-2 and short-chain fatty acid concentrations.

**Results:**

We demonstrated that inflammation, chemically or biologically induced, restructures the chemical and microbial environment of the gut over a 16-day period. We observed that the ten mice within the *Salmonella* treatment group had a variable *Salmonella* relative abundance, with three high responding mice dominated by >46% *Salmonella* at later time points and the remaining seven mice denoted as low responders. These low- and high-responding *Salmonella* groups, along with the chemical DSS treatment, established an inflammation gradient with chemical and low levels of *Salmonella* having at least 3 log-fold lower lipocalin-2 concentration than the high-responding *Salmonella* mice. Total short-chain fatty acid and individual butyrate concentrations each negatively correlated with inflammation levels. Microbial communities were also structured along this inflammation gradient. Low levels of inflammation, regardless of chemical or biological induction, enriched for *Akkermansia* spp. in the Verrucomicrobiaceae and members of the Bacteroidetes family S24-7. Relative to the control or low inflammation groups, high levels of *Salmonella* drastically decreased the overall microbial diversity, specifically driven by the reduction of *Alistipes* and Lachnospiraceae in the Bacteroidetes and Firmicutes phyla, respectively. Conversely, members of the Enterobacteriaceae and *Lactobacillus* were positively correlated to high levels of *Salmonella*-induced inflammation.

**Conclusions:**

Our results show that enteropathogenic infection and intestinal inflammation are interrelated factors modulating gut homeostasis. These findings may prove informative with regard to prophylactic or therapeutic strategies to prevent disruption of microbial communities, or promote their restoration.

**Electronic supplementary material:**

The online version of this article (doi:10.1186/s40168-017-0264-8) contains supplementary material, which is available to authorized users.

## Background

The bacterial species *Salmonella enterica* includes over 2500 serovars [1]. One of the most common causes of human gastroenteritis is serovar Typhimurium [2]. This serovar has long served as a model organism for studies of pathogenesis in murine models. Upon ingestion, *Salmonella* injects effector proteins into the host intestinal epithelial cells [3]. These effectors trigger the uptake of *Salmonella* into the host cells and initiate inflammation that disrupts the microbiota. This disruption presumably reduces competition for nutrients, and it also causes an oxidative burst that leads to the accumulation of tetrathionate, nitrate, and oxygen, all of which are used as respiratory electron acceptors by *Salmonella* [4–10]. This respiratory metabolism confers a growth advantage to *Salmonella* over the fermentative commensal bacteria, allowing this pathogen to rapidly proliferate within the intestinal microbial community.

One major issue with mouse models is that mice are highly resistant to *Salmonella-*induced inflammation, which is thought to be due to the mouse gut microbiota rather than the host itself. Either the use of germ-free mice or the disruption of the microbiota with antibiotics allows *Salmonella* to induce inflammation in most murine models [11–14]. Unfortunately, the germ-free or antibiotic-treated models are not conducive with regard to understanding how *Salmonella* alters the response of the commensal microbial community. Alternatively, it was recently discovered that the CBA/J murine model allows persistent colonization of the gastrointestinal tract by *Salmonella*, which eventually leads to inflammation approximately ten days post-infection [15, 16]. In this report, we use CBA/J mice to study the disruption of the healthy microbiota by *Salmonella*. Additionally, to begin to separate the microbiome response to inflammation alone rather than inflammation and the pathogen, we compare the pathogen-induced inflammation to chemically induced inflammation caused by dextran sodium sulfate (DSS). DSS increases the permeability of the mucosal barrier, allowing commensal microbiota to contact the epithelium and trigger an inflammatory response [17, 18].

*Salmonella*-mediated disruption of the commensal microbiota has been previously studied; however, the microbiota was characterized at a broad taxonomic level or using an antibiotic-treated mouse model [5, 7, 19, 20]. Here, we characterize the otherwise undisturbed fecal and cecal communities before and after disruption by *Salmonella* or DSS and focus on operational taxonomic unit (OTU) level responses. We measure changes in microbial community diversity and membership, as well as changes in the chemical environment and compare these responses to non-inflamed control mice. Results from this study provide an in-depth insight into *Salmonella* impacts on the gut environment. These findings may reveal new therapeutic strategies for prebiotics or probiotics for maintaining or restoring the microbiota in response to *Salmonella* perturbation [21–28]. Furthermore, examining responses of the commensal microbiota and chemical environment to inflammation has broader ramifications to other gastrointestinal diseases, including ulcerative colitis, Crohn’s disease, and colon cancer [29–31].

## Results

### Experimental design and 16S rRNA gene sequencing

**Fig. 1:**
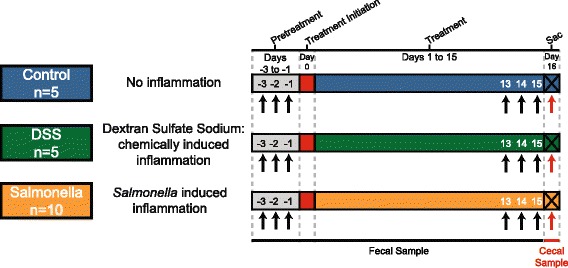
Experimental design illustrating fecal and cecal sample collection for microbial community analysis. From *left* to *right*, *gray* boxes indicate pretreatment, *red* boxes indicate treatment initiation, and *colored* boxes denote treatments (Control = *blue*, DSS = *green*, and *Salmonella* = orange). *Black arrows* indicate fecal sample collection, and *red arrows* indicate cecal sample collection

To investigate the impacts of inflammation on the gut microbial community, we performed 16S rRNA gene profiling on the microbiota of chemically inflamed and *Salmonella*-inflamed mice. We compared control mice (*n* = 5), receiving no inflammation treatment, to five mice that were administered DSS (chemically inflamed) and ten mice that were inoculated with 10^9^ CFU *Salmonella enterica* serovar Typhimurium strain 14028 (Fig. 1). Fecal samples were collected 3 days prior to day 0 (treatment) and 3 days prior to day 16 (sacrifice). Cecum samples were obtained on day 16 (Fig. 1). Cecal (*n* = 20) and fecal (*n* = 117) microbial communities were surveyed using Illumina amplicon sequencing of the 16S rRNA gene (V4 region). For fecal samples, we had 60 pretreatment samples (20 samples/day for 3 days for all treatments), 60 late treatment samples (20 samples/day for 3 days for all treatments), and 20 cecum samples (*n* = 140). Three samples over the course of the experiment yielded insufficient reads to be included in this analysis (Additional file 7). A total of 2,587,891 high-quality, classifiable reads were generated for the 137 samples. After merging reads, clustering OTUs at 97% identity, and removing chimeric sequences (see the “Methods” section), we identified a total of 6045 OTUs that were present in at least five samples. The OTU table with taxonomic assignment and the FASTA file is included (Additional files 1 and 2, respectively).

### Inflammation type (chemical or biological) structures gut microbial communities

**Fig. 2:**
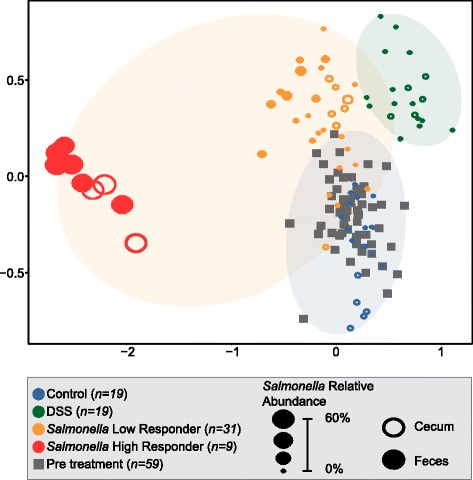
Non-metric multidimensional scaling (NMDS) showing the treatment response and designated groupings. Bray-Curtis similarity metric from the pretreatment fecal, late fecal, and cecal samples (stress = 0.08) show a statistically significant (Mrpp, *p* = 0.001) separation of microbial communities from control, DSS, low-responder, and high-responder groups at late time points. The *orange* ellipse represents the *Salmonella* treatment group (high and low responders). The legend with sample assignment is shown in the *gray* box. The sample points are sized for *Salmonella* relative abundance. *Open symbols* denote cecal, while closed denote fecal, samples

The relative similarity of microbial communities between samples (beta diversity) for all pretreatment fecal (days −3, −2, −1), late fecal (days 13, 14, 15), and late cecal samples was examined by calculating a Bray-Curtis dissimilarity matrix and visualized using non-parametric multidimensional scaling (NMDS) in two dimensions (Fig. 2). These analyses revealed that DSS and *Salmonella*-treated mice have statistically different late fecal and cecal microbial communities compared to all pretreatment and control samples (Fig. 2). Pretreatment fecal samples (regardless of experimental treatment) clustered with late control fecal samples, demonstrating the stability within the undisturbed normal gut microbiota over time.

Within the *Salmonella* treatment group, we saw a variable response, as two distinct clusters were observed on the NMDS, a finding confirmed by beta-dispersion analyses. The *Salmonella* treatment had the greatest within treatment variability compared to the other treatment groups (mean distance to centroid: Control = 0.306, DSS = 0.316, *Salmonella* = 0.470). Despite using identical inoculation concentrations and procedures, *Salmonella* relative abundance ranged from <1 to 71% in the late fecal and cecal samples. At the end of the experiment, three of the mice had much higher *Salmonella* relative abundance than the other seven mice, with this high-responder group defined as having a minimum *Salmonella* relative abundance of 49 and 46% in the late time point fecal and cecal samples, respectively. This differential *Salmonella* response was also visually verified upon autopsy, as ceca in the high responders were pus-filled, unlike the other *Salmonella*-treated mice (Additional file 3: Figure S1). Alternatively, the low responders had a maximum of 7 and 0.5% in the late time point fecal and cecal samples, respectively. This range in *Salmonella* relative abundance was represented in the NMDS clustering, with high responders (red) clustering distinctly from low responders (orange) and control (blue) (multi-response permutation procedures (mrpp), *p* value <0.05). While the latter two groups were statistically different, low responders were more similar to the control than high responders. Interestingly, this clustering was maintained when the *Salmonella* OTU was removed from the analyses (Additional file 4: Figure S2; see the “Methods” section), demonstrating that the differences observed between treatments were not attributed solely to pathogen increase, but rather the overall impacts of the pathogen on the surrounding microbial community.

Our data clearly show that mice given the same *Salmonella* dose and treatment have variable susceptibility to *Salmonella* colonization. To confirm that we did not underestimate *Salmonella* relative abundance in the low-responder group due to our sampling schedule, we subsequently sequenced all fecal time points between the pre-treatment and late samples (Additional file 5: Figure S3). *Salmonella* relative abundance in the low-responder group was not uniform over time. Across all time points in the low responders, the maximum *Salmonella* relative abundance for each mouse ranged from 0.3 to 16%. This analysis showed that *Salmonella* relative abundance was higher at earlier time points, compared to the final time points we reported initially, but more importantly confirmed the low-responder group never reached our designated high-responder *Salmonella* relative abundance (>46%). Our findings confirm that we did not miss the sampling window of elevated *Salmonella* response in the low-responder mice. For subsequent analyses, we divided the *Salmonella* infected samples into “low-responder” and “high-responder” treatment groups.

**Fig. 3:**
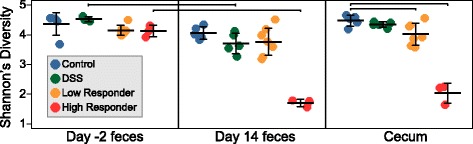
Shannon’s diversity index (H’) by treatment in day −2, day 14, and cecum samples. Strip chart displaying Shannon’s diversity by treatment in pretreatment (day −2), late (day 14), and cecum samples, with each point representing a single sample. *Horizontal black lines* show the mean, and *error bars* represent one standard deviation from the mean. Significant changes relative to pretreatment communities and control communities are denoted by *brackets* at the top of the figure

We compared alpha diversity between fecal day −2 (pretreatment), fecal day 14 (late), and cecal communities. Consistent with our NMDS pretreatment results, day −2 fecal communities had no discernable difference in Shannon’s diversity between treatment groups (Fig. 3). Comparison of fecal microbial communities from day −2 and day 14 revealed that only DSS and high-responder groups had a significant decrease in Shannon’s diversity over time, while the control was unchanged and the decrease in the low-responder group was not significant. Notably, when examining the richness over time, only the high-responder group had a significant decrease (~54%) in OTUs (Additional file 6). Unlike our fecal results, the cecal microbial communities from both low- and high-responder groups had significantly lower Shannon’s diversity compared to the control group. Our findings show that in the gastrointestinal tract, high relative abundance of *Salmonella* restructures the microbial diversity more significantly than chemical treatment (DSS).

### Inflammation and metabolites are correlated with treatment groups

**Fig. 4:**
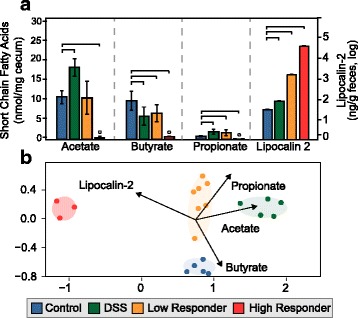
Quantification of short-chain fatty acid (SCFA) and inflammation in the gut at late time points among treatments. **a** Bar chart shows the average of SCFA (acetate, butyrate, and propionate, left *y*-axis) and Lipocalin-2 (right *y*-axis, log scale) concentrations in cecal and fecal samples with error bars representing one standard deviation. Significant changes relative to the control are denoted (*brackets*). High responders are below the limit of detection for SCFA, denoted by *small circles*. **b** NMDS of Bray-Curtis similarity metric shows a statistically significant separation of cecal microbial communities from control, DSS, low-responder, and high-responder groups (stress = 0.07). Vectors were calculated with envfit and represent statistically significant correlations of chemical data relative to microbial community data (*p* values: acetate = 0.001, butyrate = 0.003, propionate = 0.046, inflammation = 0.001)

As a measure of inflammation, we utilized Lipocalin-2, an innate immune protein induced during inflammatory responses (Fig. 4a, Additional file 7) [32, 33]. Fecal lipocalin-2 levels provide a sensitive and broadly dynamic method to monitor inflammation, specifically for low levels of inflammation [32]. All treatment groups had increased Lipocalin-2 compared to the control and were statistically different from each other (*p* value <0.01). Controls had the lowest amount of Lipocalin-2, followed by DSS, then low responders, and finally the high responders, the latter with a 3 log-fold increase in inflammation relative to the control. This indicates that inflammation level correlated to treatment group microbial communities.

Microbially produced short-chain fatty acids (SCFA) maintain the gut barrier, participate in host signaling, and contribute to host energy [34], so we were interested in the impacts of inflammation on cecal SCFA concentrations. The cumulative concentrations of acetate, butyrate, and propionate (here reported as total SCFA) in the ceca were not significantly different between the control, DSS, or low-responder groups, but were significantly decreased in the high-responder group (Fig. 4a, Additional file 6). Individual SCFA had a treatment specific response. Acetate, butyrate, and propionate were all below detection in the high-responder group, while relative to the control, the DSS and low responders had significantly decreased butyrate and increased propionate concentrations, while acetate increased significantly only in the DSS group. Across all samples, the amount of inflammation negatively correlated to the total concentration of short-chain fatty acids and to butyrate concentrations. At a more global level, changes in the chemical environment (e.g., inflammation and SCFA concentrations) corresponded to changes in cecal microbial community structure (Fig. 4b) (envfit, *p* < 0.001).

### Identifying key microbial determinants for each treatment group

To be consistent with prior reports examining the impacts of *Salmonella* colonization on the gut microbiota [5–7, 19, 20, 35], we examined our data to see if groupings observed on the NMDS (Fig. 2) were consistent with changes in membership at the class level. Control fecal microbial communities at day 14 were dominated by Firmicutes and Bacteroidetes phyla, especially within the Clostridia (62 ± 9%) and Bacteroidia (34 ± 8%) classes (Additional file 8: Figure S4). Despite differences in SCFA and inflammation levels, Clostridia and Bacteroidia relative abundance did not change significantly in the low-responders or DSS groups relative to the control. Notably, the relative contribution of these two classes was significantly decreased when *Salmonella* exceeded 46% relative abundance (e.g., high-responder group only), but was not correlated to *Salmonella* relative abundance across the experiment. Relative to the other treatments, microbial communities in the high-responder group were enriched in the Gammaproteobacteria (57%, driven largely by *Salmonella*) and the Bacilli classes (Additional file 8: Figure S4).

**Fig. 5:**
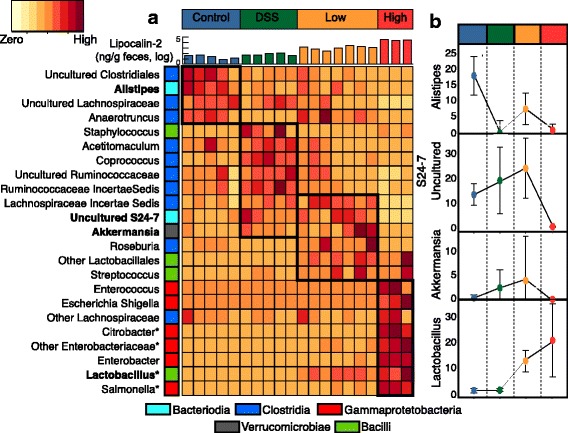
Heat map of discriminant genera determined by LEfSe analysis. **a** All taxa that were discriminant features in at least one treatment (LDA score >2) are shown, with phylogenetic class reported as *colored boxes*. Relative abundance is shown scaled by relative abundance within a genus, with a legend provided in the top left. A *bar chart* at the top of the heat map displays Lipocalin-2 concentrations in individual samples, colored by treatment. All genera are discriminant features relative to the control, while some are discriminant features relative to all treatment groups as denoted by *asterisks*. **b** The relative abundance of a single OTU representative from *Alistipes*, S24-7, *Akkermansia*, and *Lactobacillus* within each treatment were graphed by treatment with median and standard deviation shown

To more specifically resolve which genera were responsible for driving the treatment group differences, we performed linear discriminant effect size (LEfSe) analysis on day 15 fecal samples [36, 37]. For taxa with linear discriminant analysis (LDA) scores greater than 2 in at least one group, we summarize the relative abundance across the treatment groups (Fig. 5). Some of these discriminant genera had abundance patterns shared across treatments, while others were unique to a specific treatment. Within the control group, four discriminant genera were identified, including members of the Clostridia (e.g., uncultured members within Lachnospiraceae) and Bacteroidia (*Alistipes*) (Fig 5a). Of these, the *Alistipes* response was the strongest (LDA score 4.8) and driven by one OTU (AY990081). The mean relative abundance of this *Alistipes* OTU in the control group is significantly higher (17%) than the relative abundance in DSS (2%), low-responder (9%), and high-responder (2%) groups (Fig. 5b). This finding indicates *Alistipes* may be especially sensitive to inflammation and in light of our chemistry data suggests it may also be a key butyrate producer in the healthy gut.

Our findings showed that differences in inflammation type (chemical or biological) and inflammation amount (DSS and low responder compared to high responder) corresponded to altered microbial membership. In the chemical but not the biological inflamed guts, LEfSe identified six genera unique to the DSS communities including two genera within the Lachnospiraceae (*Coprococcus* and an uncultured genus) and two uncultured genera within the Ruminococcaceae. At day 15, *Coprococcus* had a significantly higher mean relative abundance in the DSS treatment group (2.2 ± 0.4%), compared to all other groups (<0.05%). These findings suggest taxa are finely tuned to tolerate the low levels of inflammation induced by the chemical DSS treatment and thus could be responsible for increased acetate production observed only in this treatment relative to the control.

The low-responder group had higher levels of inflammation relative to the control and DSS groups (Fig. 4). In the low responder communities, LEfSe identified eight genera including members of *Akkermansia, Roseburia,* and a formerly uncultivated Bacteroidetes family S24-7 (*Candidatus* Homeothermaceae or Muribaculaceae) [38, 39]. Interestingly, several of these taxa were also enriched by chemical inflammation (DSS), but were not detected in the control or high responder groups. For instance, *Akkermansia* mean relative abundance was elevated in DSS (3%) and low responders (9%) but not detected in high responders or control (<0.01%). The same response was observed for S24-7 (Fig. 5). This result suggests that these taxa respond positively to low levels of inflammation but are decreased when *Salmonella* relative abundance or inflammation is high. These taxa that co-occur across both treatments may also be responsible for increased propionate observed in both the DSS and low-responder groups,

The *Salmonella* high responders group had the most elevated levels of inflammation (Figs. 4a and 5a). Besides *Salmonella* (LDA = 5.3), LEfSe identified five significant Enterobacteriaceae genera within the Gammaproteobacteria (e.g., *Enterobacter*, *Citrobacter*, *Enterococcus*, *Escherichia/Shigella*) that distinguished the high-responder microbial communities from all other groups (Fig. 5). LEfSe also identified *Lactobacillus* as discriminant taxa in the high responders, and this genus is the primary driver of the Bacilli class response enriched in all *Salmonella*-treated mice, accounting for on average 9 and 26% in the low- and high-responder treatment groups, respectively. This response suggests that *Lactobacillus* may co-enrich with the presence of *Salmonella* and may not be responding to the high levels of inflammation.

**Fig. 6:**
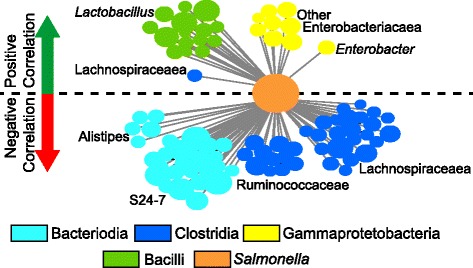
Network analysis showing OTUs with a significant positive or negative co-correlation to *Salmonella*. Each *line* represents a correlation between a single OTU and *Salmonella* relative abundance (all *p* values <0.05). *Circles* are sized by log relative abundance and colored by class, with the exception of *Salmonella* (*orange*—center node). OTUs above the *dashed black line* are positively correlated to *Salmonella* (*green arrow*), while OTUs below are negatively correlated (*red arrow*)

To verify that the trends observed in the *Salmonella* treatment were also observed in the cecal samples, we performed a Weighted Gene Correlation Network Analysis (WGCNA). This approach examined OTUs that were positively and negatively statistically correlated to *Salmonella* relative abundance (Fig. 6). Similar to our fecal data, the relative abundance of the *Lactobacillus* and Enterobacteriaeceae were positively correlated to *Salmonella*, while multiple OTUs within the family Lachnospiraceae, S24-7, and *Alistipes* were negatively correlated. These analyses also demonstrated the value of examining responses at the OTU level, and not broader taxonomic levels (e.g. class), as a single Lachnospiraceae OTU was strongly positively correlated to *Salmonella* abundance.

## Discussion

### Principal findings of the study

Here, we investigated the impacts of *Salmonella* expansion in the mouse gastrointestinal tract on the commensal microbial community and the chemical environment. To our knowledge, this is the first study to directly compare and contrast DSS-induced chemical inflammation with *Salmonella*-induced inflammation. Our principle findings are the following: (1) the extent of *Salmonella* colonization in the mouse gastrointestinal tract is variable; (2) gut microbial community membership is congruent with *Salmonella* relative abundance and inflammation level; (3) commensal *Alistipes* and Lachnospiraceae taxa decrease along an inflammation gradient; (4) low levels of inflammation (regardless of source) increase the relative abundance of *Akkermansia* and S24-7; and (5) *Lactobacilli* and members of the Enterobacteriaceae are co-enriched with *Salmonella*.

### Mice have differential *Salmonella* susceptibility

Here, we report infected mice have a large range in *Salmonella* colonization relative abundance (<1 to 71%), with three of the ten mice classified as high (>46%) responders. This variation was shared between cecal and fecal samples, demonstrating it was not an artifact of gastrointestinal tract sampling location. We did verify that timing had a negligible effect on *Salmonella* relative abundance, as some of the low responders had increased abundance at earlier time points (0.25–16%) relative to the last time points we sampled (0.25–7%). Importantly, however, none of the low-responder mice ever had sufficient *Salmonella* relative abundance to be considered a member of the high-responder group; thus, the groupings likely reflect the unique chemical and biological environments caused by different amounts pathogen induced inflammation.

While we recognize our high responder sample size is limited (*n* = 3), similar to our findings, others have hinted at this variation (and also recovered high responders) in *Salmonella* relative abundance in infected mice. Our findings expand upon prior studies in several ways [5–7, 19, 20, 35]. First, most prior studies used a “streptomycin mouse model,” where antibiotics are given prior to *Salmonella* inoculation, thereby confounding whether the observed *Salmonella* relative abundance variation was due to an altered initial microbial community or *Salmonella* colonization effectiveness. In contrast, the CBA/J mouse model does not require antibiotic treatment prior to inoculation; thus, our *Salmonella* treated mice had highly similar initial microbial community membership and structure (Fig. 2; Bray-Curtis similarity >93% for pre-treatment communities), despite clear differences in the terminal communities.

Second, the variability in *Salmonella* relative abundance may not have been emphasized previously due to differences in data reporting, as studies often report the average *Salmonella* relative abundance in the terminal sample for the entire treatment. For instance, reporting our fecal data in that fashion would result in an average *Salmonella* relative abundance of 19%, absolutely consistent with prior reports of ~25%, but obscuring the true variation. Analysis of published data sets with the same mouse model (*n* = 5), but using the high-responder criterion established here (>46%), revealed a high responder rate of 33% [5], which was nearly identical to the 30% observed here (*n* = 10). Here, we are the first to quantify *Salmonella* infection efficiency and report differential chemical and microbial responses caused by variability. Future quantification of pathogen relative abundance can better inform investigations on the host and microbiome mechanisms underlying *Salmonella* resistance, a feature also commonly reported in humans [40–42].

### Inflammation gradient correlates with SCFA and microbial community profile

Hosts rely on their gut microbiota to produce SCFA for energy, with acetate, propionate, and butyrate being the most abundant. Furthermore, SCFA are often implicated as regulators for intestinal inflammation. Many human and animal studies show decreased SCFA concentration with intestinal inflammation including instances of enteropathogenic infections, colitis, and irritable bowel syndrome [43–45]. Here, we also show the SCFA types (acetate, butyrate, propionate), and overall total concentrations are altered by changes in inflammation amount and source (biological and chemical). These changes in SCFA corresponded to differences in microbial membership across treatment groups.

The chemical environment of the gut was reflected in the microbial community, as the inflammation gradient and SCFA concentrations were significantly related to microbial community sample clustering. The most obvious changes in SCFA occurred in the highly inflamed *Salmonella* gut, where SCFA were significantly depleted relative to the non-inflamed control. Consistent with prior reports in germ-free mice [45], decreased SCFA levels may feedback to further exacerbate inflammation, contributing to the 3 log-fold higher lipocalin-2 levels detected in our high responder *Salmonella* samples.

We consider several possibilities for the drastically decreased SCFA in the high-responder group. First, it is possible that SCFA are not produced due to significant remodeling of the microbial community (decreased richness and membership of commensal taxa). Baumler and colleagues first reported that the relative abundance of commensal taxa (especially members of the class Clostridia) and butyrate decreased with *Salmonella* infection [5], and this decreasing butyrate [5], as well as reactive oxygen species from inflammation, provided alternative electron acceptors like oxygen, nitrate, and tetrathionate that allow for Salmonella expansion in the gut [9]. Here, we extend the findings to include decreased acetate and propionate co-occurring with *Salmonella* expansion and Clostridia reduction in the high-responder group. Furthermore, our low responder and DSS groups shed new light on the links between *Salmonella* and butyrate. For example, in the DSS and low-responder groups, butyrate decreased significantly from the control, while there was no significant change in Clostridia class level relative abundance, showing that this response is due to inflammation (DSS or *Salmonella* induced) and not just the pathogen presence. A second explanation for the decreased SCFA in the high responders is that these mice may have consumed less food, yielding less SCFA. A third explanation for below detectable SCFA only in the high responder group is that SCFA may be rapidly consumed by *Salmonella* and other respiratory Enterobacteriaceae taxa stimulated by presence of alternative electron acceptors produced during high inflammation levels [9, 10, 43]. Our findings demonstrate the need for time series metatranscriptomic studies that account for butyrate production and consumption activities in light of inflammation and pathogen expansion.

### Microorganisms depleted by inflammation

Given the class Clostridia contains over 12 families with considerable OTU richness (457,466 OTUs), and broad metabolic diversity [46–48], we examine the relationship between *Salmonella* and Clostridia at more resolved taxonomic levels (genus, OTU). For instance, within the Clostridia class, different Lachnospiraceae OTUs were enriched in each treatment (high, low, and DSS), with one OTU even increasing in response to *Salmonella* (New.ReferenceOTU56). Consistent with the differential response of certain members of the Lachnospiraceae across treatments, the genomic potential of the known isolates is also diverse. Of the 69 isolate Lachnospiraceae genomes, 82 and 97% encode the capacity for butyrate and acetate production, respectively. Together, these findings demonstrate that moving forward, OTU level changes should be considered, as members of the class Clostridia are present in all treatments, but strains are niche differentiated enabling unique biological and chemical treatment responses.

In addition to response of specific Clostridial members, our results show that a single dominant *Alistipes* OTU, a member of the Bacteroidia, decreased significantly in all defined groups relative to the control at day 15 (Fig. 5). This OTU is a defining feature of the non-inflamed microbial community, and given the inclusion of a DSS chemical inflammation treatment, we can clearly show that this response is not necessarily to *Salmonella* but rather inflammation. *Alistipes* depletion in inflamed gut environments has been demonstrated in humans with liver disease and in murine models for colitis [49–51]. We show a correlation between decreasing *Alistipes* relative abundance and decreasing butyrate concentrations, findings that may be attributed to end products of *Alistipes* metabolism. We mined publically available *Alistipes* isolate genomes and found that 14 out 14 genomes contain the capacity for butyrate production via butyrate kinase (see the “Methods” section). It is also possible that *Alistipes* produce butyrate from amino acids, as metagenomic studies have shown that members of the *Alistipes* have the capacity to ferment lysine to produce butyrate [52, 53]. We also mined publically available *Alistipes* genomes for this capacity and found that 14% of *Alistipes* genomes have the entire pathway to produce butyrate from lysine (see the “Methods” section for pathway). Furthermore, isolate studies show succinate as a significant end product of *Alistipes* metabolism which may stimulate butyrate production by other commensal microorganisms in the gut through the succinate pathway [54, 55]. While our *Alistipes* OTU is <95% similar to 16S sequences in isolate genomes, our findings taken together with *Alistipes* genomic evidence and *Alistipes* isolate studies suggest that future *Salmonella* work should be expanded beyond Clostridia to examine the contribution of *Alistipes* to maintaining host homeostasis.

### Known mucin-degrading microorganisms are enriched by low-level inflammation

Our experimental design allowed us to investigate the response to inflammation amount regardless of the causative agent (e.g., chemical, pathogen), with low inflammation levels represented by the DSS and low-responder groups. It has been documented that inflammation upregulates a dose-dependent host response, such that lower levels stimulate mucin production while high levels inhibit mucin production [56, 57]. Mucin is the major protective component in the gastrointestinal epithelium, providing a barrier between human epithelial cells and invading pathogens. *Akkermansia,* a member of the Verrucomicrobia, is known to degrade mucin as its sole carbon and nitrogen source [58]. Consistent with its role as a mucin degrader, *Akkermansia* is increased from controls only during treatments with low levels of inflammation, when mucin may be produced. In addition, recent genomic evidence also suggested members of the family S24-7 have similar mucin degradation capacity to *Akkermansia*, perhaps explaining the co-occurrence pattern of these two taxa in our low inflammation treatments [37]. Our findings suggest it is the amount (low not high) of inflammation not the source that dictates *Akkermansia* and S24-7 relative abundance, findings which may explain the lack of congruence between inflammation and *Akkermansia* in the literature today [58–62].

Another interesting finding from our study is that propionate concentrations increased in both the low-inflammation treatments. Given the enrichment of *Akkermansia* and S24-7 in both these low-inflammation treatments, we mined publically available genomes for propionate production. Of the three known pathways for propionate production [63], the pathway that proceeds through succinate via methylmalonyl-CoA decarboxylase (E.C. 4.1.1.41) is the most prevalent in these genomes. Both *Akkermansia* genomes and the recently published genomes within the family S24-7 encode the capacity for propionate production [38]. This increased propionate production may be a positive feedback on inflammation, as recent reports have suggested propionate stabilizes inflammation in the gut [64, 65]. Our findings provide insight into possible microbial probiotics that may enhance propionate stabilization through addition or stimulation of key taxa found here, including *Akkermansia* and members of S24-7.

### Bacterial taxa that benefit from *Salmonella*-triggered inflammation

We show that specific members of the Proteobacteria are exclusively enriched when *Salmonella* relative abundances exceed 46% (high responders, *n = 3*). Co-enrichment of members of the family Enterobacteriaceae with *Salmonella* infection has also been reported in several other mouse models [5, 7, 10, 20, 66]. Additionally, a study conducted on feces from human patients with *Salmonella* gastroenteritis reported an increase in Enterobacteriaceae including *Citrobacter*, which was one of the most abundant members in our study [35]. These findings suggest that high amounts of *Salmonella* and concurrent high inflammation levels induce specific microbial community changes different from low inflammation levels, which favors the expansion of closely related organisms [7, 66].

Our findings also show enrichment of *Lactobacillus* within the *Salmonella* (low and high) treatment. *Lactobacillus* is generally used as a probiotic for enteric infections, as some of these organisms secrete compounds that inhibit *Salmonella* virulence factors and motility [67]. To further investigate if *Lactobacillus* increased with *Salmonella* in other studies, we mined 16S rRNA reads that were publically available from studies with the same mouse model as ours [5]. Interestingly, we found enrichment of two *Lactobacillus* OTUs from the colon of *Salmonella*-infected mice. Similarly, in humans with enteric infections including *Salmonella*, *Lactobacillus* increased in infected patients [34, 35]. While the *Lactobacillus* OTUs are not shared across these studies, this finding suggests the relationship between *Salmonella* and *Lactobacillus* may be significant. One possibility is that the *Lactobacillus* members enriched during *Salmonella* infection are functionally distinct from the strains used as probiotics. Alternatively, the enrichment of *Lactobacillus* may be what eventually helps to eliminate *Salmonella*, consistent with the efficacy of probiotics [68, 69].

## Conclusions

Enhancing our understanding of how intestinal microbial communities change in response to inflammation is critical to managing a multitude of diseases including enteric infection, Crohn’s disease, irritable bowel syndrome, and colon cancer. Here, we report microbial and chemical changes in the host gut environment in response to DSS- and *Salmonella*-induced inflammation, in order to distinguish between changes caused by enteropathogenic takeover and those caused by intestinal inflammation. While several studies have also shown that *Salmonella*-induced inflammation causes an enrichment of *Enterobacteriaceae* and depletion of Clostridia [5, 7, 9, 35], these identifications were at a broad taxonomic level. The effects of enteropathogenic expansion and the consequences of host inflammation on the intestinal microbiota are only beginning to be elucidated. Further metagenomic studies with strain-resolved information and paired transcript data are required to understand how the key taxa presented here are enhanced or diminished in response to the unique chemical environment created by increased *Salmonella* biomass.

## Methods

### Strains and media

*S. enterica* serovar Typhimurium strain 14028 (*S.* Typhiumurium 14028) was grown in Luria-Bertani (LB) broth in a roller drum at 37 °C overnight. For inoculation, the overnight culture was washed and resuspended in water.

### Animals and experimental design

Female, age-matched (6 to 10 weeks old) CBA/J mice were purchased from Taconic Farms, Inc. Animals were housed in groups of five by treatment (Control = 1 cage of 5, DSS = 1 cage of 5, and *Salmonella* = 2 cages of 5) and were fed ad libitum Harlan mouse chow (mean 16% protein, 5% fat, 3.5% crude fiber).

Mice in the control group (*n = 5*) did not receive any treatment throughout the experiment. Concurrent with controls, experimental inflammation treatments, DSS (dextran sulfate sodium) (abiotic inflammation) and *Salmonella* (biotic inflammation), were run for 16 days. Mice in the DSS group (*n = 5*) received 4% DSS in drinking water the entire duration of the experiment. We selected DSS as a control to our pathogen-induced inflammation, as it is commonly used to initiate an inflammatory response [18] and has been shown that DSS is not a substrate that supports growth of intestinal microflora [70]. Mice in the *Salmonella* group (*n = 10*) were orally inoculated with 10^9^ CFU of a washed overnight culture of *S. typhimurium* 14028 on day 0 with no subsequent treatment. This animal experiment was performed using protocols approved by The Ohio State University Institutional Animal Care and Use Committee (IACUC; OSU 2009A0035).

### Sample collection

Fecal samples were collected from all mice for three consecutive days prior to treatment on day 0 and prior to sacrifice on day 16. Fecal pellets were collected from each mouse on autoclaved aluminum foil. Fecal pellets were immediately transferred to pre-labeled microcentrifuge tubes, flash frozen in liquid nitrogen, and stored at -80 °C until further processing. Cecal samples were harvested from all mice on day 16 post-treatment, flash frozen in liquid nitrogen, and then stored at −80 °C until further processing.

### DNA extraction and sequencing

Total nucleic acids were extracted using the PowerSoil DNA Isolation kit (MoBio), eluted in 100 μl of elution buffer provided, and stored at −20 °C until sequencing. DNA was submitted for sequencing at Argonne National Lab at the Next Generation Sequencing facility using Illumina MiSeq with 2 × 251 bp paired end reads following established HMP protocols [71]. Briefly, universal primers 515F and 806R were used for PCR amplification of the V4 hypervariable region of 16S rRNA gene using 35 cycles. The 515F primer contained a unique sequence tag to barcode each sample. Both primers contained sequencer adapter regions.

### 16S rRNA data processing

Data processing was performed using QIIME 1.9.0, with specific processing steps as follows [72, 73]. Briefly, raw fastq data were demultiplexed and quality filtered to a Phred score of 20. OTUs were chosen in a two-step process. First, sequences were clustered into OTUs using UCLUST followed by de novo OTU picking. OTUs were checked for chimeras using RDP gold database and assigned taxonomy using the 97_SILVA_111 rep set [74]. Sequences were used for comparison of the relative abundance of OTUs in at least five samples. A total of 6078 OTUs were found when using at least one sample and did not alter beta diversity of samples, as the NMDS had all of the same attributes (stress, ANOSIM, mrpp, betadispersion). Raw reads were deposited on NCBI under bioproject PRJNA348350 (pending). The final OTU table and a fasta file for above methods are included (Additional files 1 and 2). Raw reads from Chavez et al*.* were downloaded from NCBI and processed as above.

We inferred metabolic capacity of key taxa identified in our 16S rRNA analysis by using publically available genomes. Specified genes (e.g., methylmalonyl-CoA decarboxylase, butyrate kinase) were queried to each genome using BLASTp [75]. Genomes were accessed via NCBI and JGI-IMG (analysis performed with data from December 2016) [75, 76]. *Alistipes* genomes were mined for the lysine pathway: lysine-2,3-aminomutase (EC 5.4.3.2), lysine-5,6-amino mutase (alpha and beta, EC 5.4.3.4), 3,5-diaminohexanoate dehydrogenase (EC 1.4.1.11), 3-keto-5-aminohexanoate cleavage enzyme (EC 2.3.1.247), 3-aminobutyryl-CoA ammonia-lyase (EC 4.3.1.14), butyryl-CoA dehydrogenase (EC 1.3.99.2), and butyryl-CoA:acetoacetate CoA-transferase beta subunit (alpha and beta, EC 2.8.3.9).

### Statistical analyses

Alpha diversity of microbial communities was calculated using the diversity function, with richness and Shannon’s diversity (H’) used as the indices [77, 78]. To analyze beta diversity among samples, analysis of Bray-Curtis dissimilarities was calculated using the relative abundance of OTUs and was visualized using non-parametric multidimensional scaling (NMDS) with R (ggplot package). The goodness of fit for the data was determined by the stress of the non-parametric fit [78, 79]. Significance of community composition differences among classified sample groups was determined by analysis of similarities (ANOSIM) and mrpp) [77, 79]. For analysis of beta diversity without the *Salmonella* OTU (Additional file 4: Figure S2), the dominant *Salmonella* OTU was removed from Additional file 1 and relative abundance was recalculated for each OTU within each sample by normalizing to the remaining sample relative abundance. An NMDS was generated as described above with this new OTU table. Beta dispersion was calculated using the betadisp command with vegan package in R, while significance of dispersion was tested via anova. For all R commands regarding diversity measures, see Additional file 9. All above analyses were done using the vegan package in R.

Day 15 fecal samples were analyzed using linear discriminant analysis effect size (LEfSe) [36]. Linear discriminant effect size (LEfSe) analysis was performed at the genus level to find features (genera) differentially represented between defined groups. DSS, low-responder, and high-responder groups were compared individually to the control, and all groups were compared together. LEfSe combines the standard tests for statistical significance (Kruskal-Wallis test and pairwise Wilcoxon test) with linear discriminate analysis [36]. It ranks features by effect size, which puts features that explain most of the biological difference at top. LEfSe analysis was performed at the *α* value of 0.05 for the Kruskal-Wallis test and the threshold of 2 on the logarithmic LDA score for discriminative features. A heat map of discriminant features was generated using heatmap function in the statistics package in R. Side panels of key taxa were generated in R. All R scripts used to generate Fig. 4 are included as an Additional file 9.

Weighted correlation network analysis (WGCNA) was used to generate OTUs that were positively and negatively correlated to the *Salmonella* OTU among day 15 samples in the *Salmonella* treatment group (high and low responders) using the WGCNA package in R [80]. A Kruskal–Wallis test was used to compare the relative abundance of distinct taxonomic units and chemical metadata between treatment groups. Significance claimed in the text refers to a *p* value less than 0.05, unless noted otherwise. We used false discovery rate (FDR) adjusted *p* values to control for multiple comparison false positives, data included in Additional file 10 [81].

### Inflammation marker quantification

Lipocalin-2 levels were measured in fecal sample supernatants using the Duoset murine Lcn-2 ELISA kit (R&D Systems, Minneapolis, MN). This method has been shown to detect low-grade inflammation and severe colitis, and compared to histopathology, lipocalin is more sensitive for detecting low-grade inflammation [32, 33]. Briefly, frozen fecal samples were reconstituted in PBS containing 0.1% Tween 20 (100 mg/ml) and vortexed for 20 min. This homogenous fecal suspension was then centrifuged for 10 min at 12,000 rpm and 4 °C.

### Short-chain fatty acid quantification

To quantify short-chain fatty acids (SCFA), scraped cecal contents from five mice in the control group, five in the DSS group, and ten in the *Salmonella* infected group were collected and stored at −80 °C. After being lyophilized, cecal content dry weights were recorded and contents were ground on ice. Although lyophilization may reduce the total SCFA content, we selected this method to ensure equal loading of dry weight. Additionally, we conservatively report the relative SCFA amounts in one treatment group versus another rather than absolute values. Cecal contents with 3.8 to 8 mg dry weight for different samples were each transferred into a new 1.5-ml centrifuge tube, followed by the addition of 500 μl chilled methanol (Fisher Scientific) and 300 μl water (Fisher Scientific) spiked with 1.6 nmol [^13^C]-F-Asn. After being vortexed and centrifuged at 14,800 g for 1 h, the supernatant was aliquoted into four new 1.5-ml centrifuge tubes, frozen and lyophilized. An aliquot of each sample was re-suspended in 100 μl water, followed by filtration with a 0.2-μm PTFE filter (Thermo Scientific). A chemical derivatization was performed based on a protocol from Han et al*.* Twenty microliters of the solution was sequentially mixed with 10 μl of 10 mM 3-nitrophenylhydrazine (3NPH) (Sigma-Aldrich) and 10 μl of 6 mM *N*-(3-dimethylaminopropyl)-*N’*-ethylcarbodiimide (EDC) hydrochloride (Thermo Scientific) with 0.3% pyridine (Sigma-Aldrich). The reaction system was incubated at 40 °C for 2 h and cooled on ice for 1 min before dilution with 60 μl water. The solution was further diluted ten times with water before being injected for liquid chromatography-mass spectrometry analysis. A nanoACQUITY Ultra Performance Liquid Chromatography (UPLC) system (Waters, Milford, MA, USA) with a UPLC HSS T3 column (Waters, 75 μm × 100 mm, 1.8 μm) was coupled to a triple quadrupole mass spectrometer (Waters Xevo TQ-S) for SCFA analysis. Buffer A, 0.1% formic acid (Thermo Scientific) in water with 10% acetonitrile (Fisher Scientific), and buffer B, 0.1% formic acid in acetonitrile (Fisher Scientific), were used as mobile phases for gradient separation, which started with 100% A for 1 min at a flow rate of 0.8 μl/min and then followed by gradient: 1–5 min, 100–50% A; 5–7 min, 50–0% A; 7–10 min, 0% A; 10–12 min, 0–100% A; 12–30 min, 100% A. The mass spectrometer was operated in positive ion nano-electrospray ionization mode (nano-ESI+) with a capillary voltage of 3 kV, source temperature 70 °C, cone voltage 2 V and source offset 2 V. The gas flow rate for the collision cell was 0.15 ml/min. Transitions m/z 196 → 137, m/z 210 → 137, and m/z 224 → 137 with collision energy 20 eV were used for quantification of acetate, propionate, and butyrate, respectively, in multiple-reaction-monitoring mode. Another transition for each acid (m/z 196 → 138, m/z 210 → 138, m/z 224 → 138, respectively) was used for validation of quantification of acetate, propionate, and butyrate, respectively, and gave values within 14% of the first set of transitions. Standard curves were made by spiking derivatized acetic acid (Fisher Scientific), propionic acid (Acros Organics), and butyric acid (Acros Organics) into 1000-time diluted pooled cecal content extraction solutions from the high responder group and running the same LC-MS/MS analysis. Skyline-daily (v 3.5, MacCoss Lab, Department of Genome Sciences, University of Washington, Seattle, WA, USA) was used for calculating the peak area of transitions [82].

## Additional files

Additional file 1: OTU table of all samples with taxonomic assignment. (CSV 3174 kb)

Additional file 2: Sequences of all OTUs in fasta format. (FNA 10560 kb)

Additional file 3: **Figure S1.** Pictures showing the cecum of one low responder and one high responder. Pictures are denoted by outline color, with orange representing the low responder group and red representing the high responder group. Black arrow indicates the pus-filled area described in the text and was only visually present in the cecum from the high-responder group. (PDF 124 kb)

Additional file 4: **Figure S2.** Non-metric multidimensional scaling (NMDS) ordination of all samples without *Salmonella* OTU. A NMDS of Bray-Curtis similarity metric among microbial communities in each pretreatment fecal, late fecal, and cecal sample (stress = 0.10) shows a statistically significant (mrpp, *p* < 0.001) separation of cecal microbial communities from control, DSS, low-responder, and high-responder groups. Each point represents one sample with colors denoting treatment. (PDF 136 kb)

Additional file 5: **Figure S3.** *Salmonella* relative abundance through time in low-responder group. A line graph depicts *Salmonella* relative abundance through time for each low responder mouse. To better see the trends, Mouse 1 was put on a second, larger axis (right), while all other mice are scaled to the smaller axis (left). (PDF 352 kb)

Additional file 6: Mapping file detailing time point, treatment, Shannon’s diversity and richness for each sample. (CSV 5 kb)

Additional file 7: Mapping file of metadata detailing Lipocalin-2 and SCFA concentrations by mouse. (CSV 1 kb)

Additional file 8: **Figure S4.** Microbial communities of day 15 fecal samples. Stacked bar chart representing day 15 fecal microbial communities by class of *Salmonella*-treated mice, with each bar representing one mouse. Defined groups are distinguished from high at the bottom of the bar chart. (PDF 355 kb)

Additional file 9: All R commands used. (R 5 kb)

Additional file 10: FDR adjusted p-values. (CSV 88 kb)
