# Supplementary figures and images for: Chemical and pathogen-induced inflammation disrupt the murine intestinal microbiome
Source: Microbiome. 2017 Apr 27;5:47. doi: 10.1186/s40168-017-0264-8 (PMC5408407; doi:10.1186/s40168-017-0264-8)

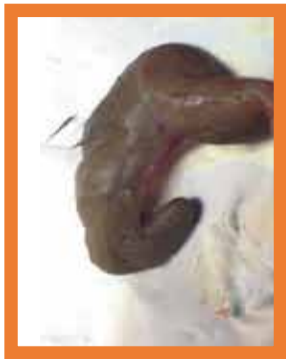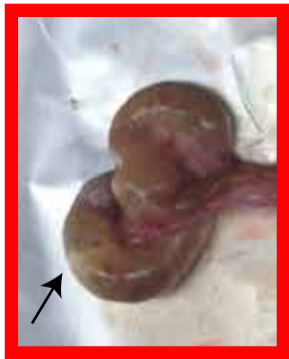

Supplement: Supplementary file 3 — Figure S1. Pictures showing the cecum of one low responder and one high responder. Pictures are denoted by outline color, with orange representing the low responder group and red representing the high responder group. Black arrow indicates the pus-filled area described in the text and was only visually present in the cecum from the high-responder group. (PDF 124 kb) [file 40168_2017_264_MOESM3_ESM.pdf]

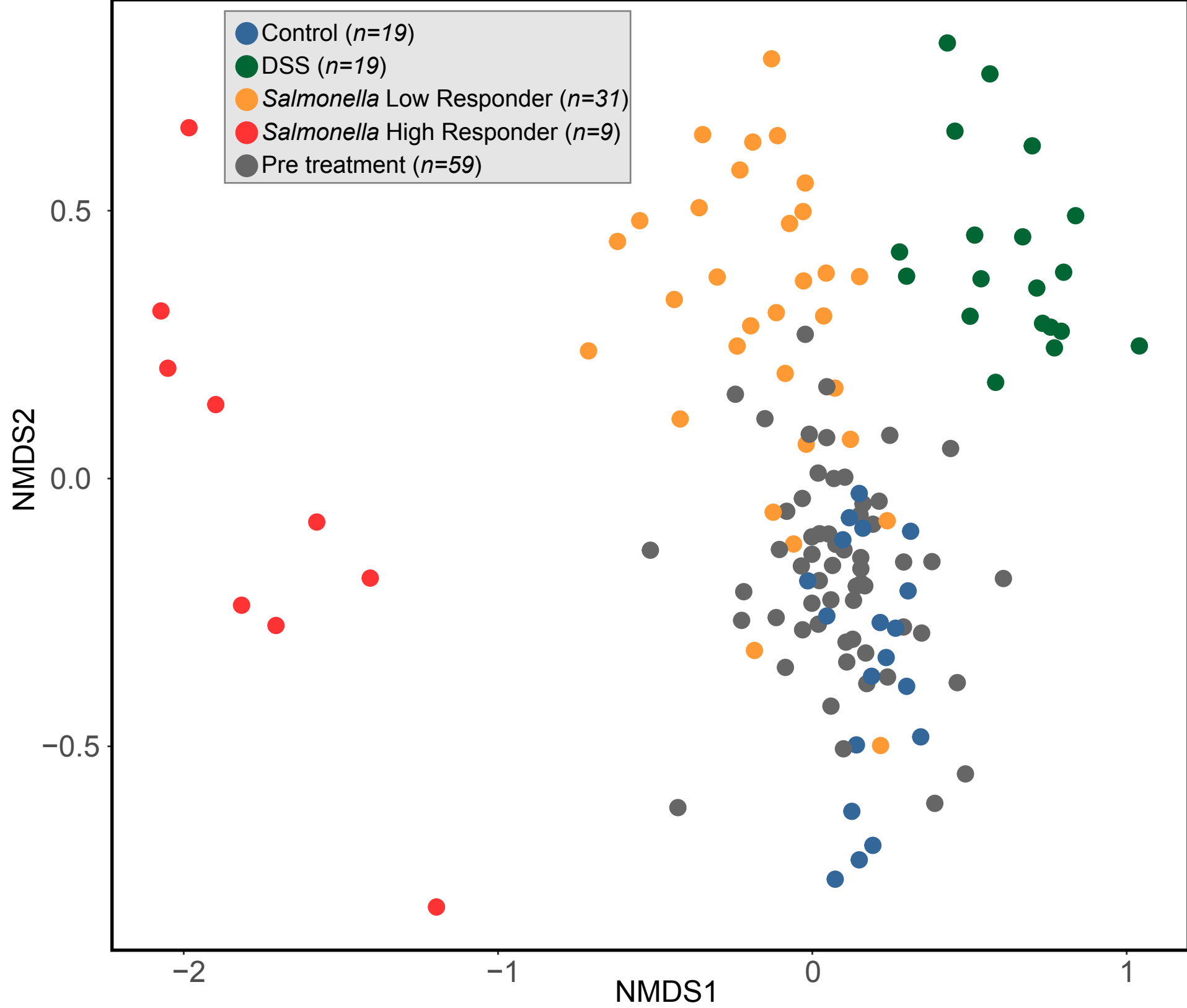

Supplement: Supplementary file 4 — Figure S2. Non-metric multidimensional scaling (NMDS) ordination of all samples without Salmonella OTU. A NMDS of Bray-Curtis similarity metric among microbial communities in each pretreatment fecal, late fecal, and cecal sample (stress = 0.10) shows a statistically significant (mrpp, p < 0.001) separation of cecal microbial communities from control, DSS, low-responder, and high-responder groups. Each point represents one sample with colors denoting treatment. (PDF 136 kb) [file 40168_2017_264_MOESM4_ESM.pdf]

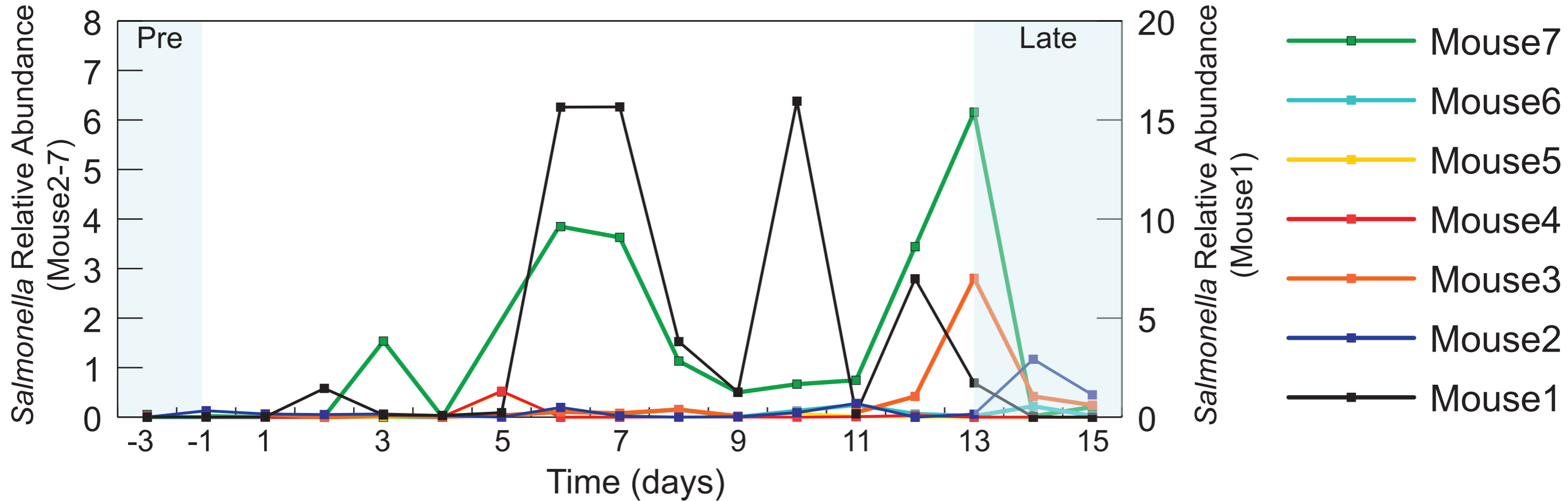

Supplement: Supplementary file 5 — Figure S3. Salmonella relative abundance through time in low-responder group. A line graph depicts Salmonella relative abundance through time for each low responder mouse. To better see the trends, Mouse 1 was put on a second, larger axis (right), while all other mice are scaled to the smaller axis (left). (PDF 352 kb) [file 40168_2017_264_MOESM5_ESM.pdf]

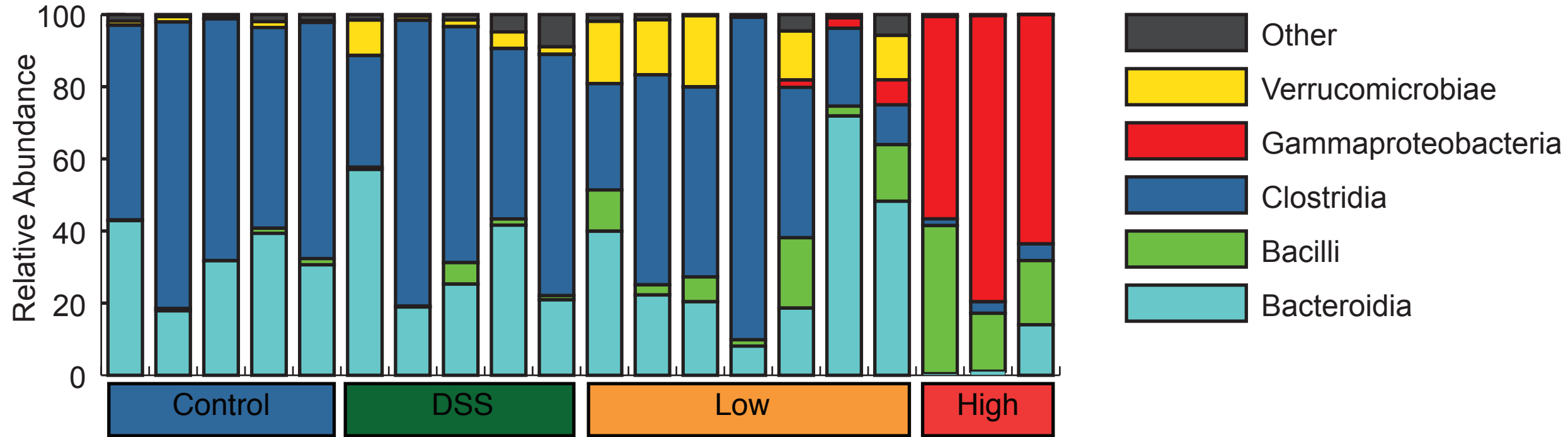

Supplement: Supplementary file 8 — Figure S4. Microbial communities of day 15 fecal samples. Stacked bar chart representing day 15 fecal microbial communities by class of Salmonella-treated mice, with each bar representing one mouse. Defined groups are distinguished from high at the bottom of the bar chart. (PDF 355 kb) [file 40168_2017_264_MOESM8_ESM.pdf]
